# Is Marriage a Sin?

**Published:** 1890-07-19

**Authors:** 


### The Hospital

A Weekly Journal of

Science, Medicine, Nursing, and Philanthropy.

*For Hospitals, Asylums, Medical Practitioners, Students, Nurses, Families, Parishes, Congregations, and the Charitable Public.*

Vol. VIII.—No. 199.] [July 19, 1890.

### Is Marriage a Sin?

Count Tolstoi has a good many admirers in this country. In the *Universal Review* the Count has pronounced against marriage on the ground that it is unchristian. If the fact be indeed so, then the days of Christianity are numbered; for it is certain that so long as man is man and woman is woman marriage both will, and ought to be, the relation of the sexes. Here are Count Tolstoi's words: "Now there is not and cannot be such an institution as Christian marriage, just as there cannot be such a thing as a Christian liturgy, nor Christian teachers, nor Church fathers, nor Christian armies, Christian law courts, nor Christian states. This is what was always taught and believed by true Christians of the first and following centuries. A Christian's ideal is not marriage but love for God and for his neighbour. Consequently in the eyes of a Christian sexual relations in marriage not only do not constitute a lawful, right, and happy state as our society and our churches maintain, but, on the contrary, are always a fall, a weakness, and a sin. Such a thing as Christian marriage never was and never could be."

Some of the friends of Christianity, it seems to us, are bent upon destroying their faith; and that by the simple but effectual process of making it incredible. Canon Liddon, who, though a pronounced High Churchman, has innumerable friends and admirers in every church in the civilized world, and among people of no church at all, has taken a stand in opposition to modern criticism which, if he were a man of science would compel him to renounce either his science, or his Christianity. A large and venerable section of the church plies us with all sorts of modern miracles for which there is no scientific evidence, and which are often in themselves as ridiculous as they are impossible of belief. And now here is Count Tolstoi, who is neither an Anglican nor a Catholic, but a kind of primitive Christian, telling us that one object of Christianity is to abolish marriage and thereby to abolish humanity, and that within little more than half a century.

Now, medical science has a right to be heard on a subject of this kind. We join issue with Count Tolstoi at once; and if we could believe that he is correctly representing the Christian faith in his utterances on marriage we should join issue with Christianity too. Science has this great and transcendant merit, that she never denies or explains away a fact. A fact is to her what a twenty feet deep lake is to a man who cannot swim; it is a reality which neither logic, nor poetry, nor any other power possessed by the man can get rid of. The water is there; he is floundering in it; and if some good samaritan does not fish him out he will be drowned as infallibly as that the day breaks when the sun rises.

Man, as he is, with all his parts and passions, is an undoubted fact. How came he to be fact? Did he make himself? And if he did not make himself, is he responsible for being the kind of creature that he is? Man was made by some power. We do not here say by what power; but we say, and no philosophy can contradict it, by some power. Who, then, is responsible for man's being as he is or what he is? The maker or the made? To say that man is responsible is to hold the chair responsible instead of the cabinet maker. The being, or the power, who formed man must alone bear the responsibility of having made him what he is. Our Christianity forbids us to dare even for a moment to think that the Divine Maker of man would wish to put any responsibility upon the creature which properly belongs to the Creator.

But the next question that arises is this: if a divine power made man as he is, was it wise and right to make him so? If it was wise and right to make him so, then clearly it must be both wise and right for man so to live and act as he was designed by his maker for living and acting. Man was made capable of love; and therefore he must love. Woman was made capable of love; and, therefore, she must love. The passion of pure love, as it exists between a natural man and an unsophisticated woman, is the most purifying and elevating of all the passions of which humanity is capable. That mutual passion finds its natural perfection in marriage: and if anything can be more blessed in the mortal state than a union of perfect mutual love, it is the birth of children and the divine love of parenthood.

There are, and always have been, certain persons who either do feel, or affect to feel, that love and marriage have something that is base in them. No words can convey to readers the scorn that at least one man feels for the innate feebleness of intellect or the prurient filthiness of heart that sees anything lower than what is noble in the relation of husband and wife, and in the facts of paternity and maternity. To cry out against these is not only to cry out against the whole natural world, as it has existed for innumerable ages, but it is to cry out against God, who, before He put forth His hand to fashion man and beast, purposed in His own mind that the beasts should be miracles of skill, and that man should be an image of Himself.

Are there not obstacles enough in the way of the acceptance of a pure and beautiful religion in these times, without searching in the dreams of the cloister for unpractical theories and counsels of perfection which are perfection only to diseased and distorted minds? Men should look deeper. If Count Tolstoi be right, nothing is possible to the reasoning man but atheism complete and final. Is it conceivable by the man who has apprehended the simple fact that to pure intellect neither time nor space exist—is it conceivable that a few thousand years ago the Divine wisdom created man and gave him the command to "increase and multiply, and replenish the earth and subdue it," and that now, without having changed man's nature at all, He bids him cease from multiplying, and sets before him the voluntary extermination of his own race as a duty? It is not conceivable. If there is to be continuity of purpose anywhere in all this vast universe, it must surely be in the central intellect and energy which created all, and which must guide all to an appointed and final destiny.

We have not considered whether Christianity does or does not teach that marriage is a sin. That is no part of our business. We may, however, in passing, express the emphatic conviction that there is not, either in the New Testament or in the Old, the smallest particle of support for the general proposition, that marriage is a sin. But of this we are assured that if Christianity does teach such an unnatural and impossible doctrine, it is not man that will cease and determine, but Christianity. Is Count Tolstoi, and are his admirers, prepared for this? If they are not, they will do well to go back to the sanctums where they evolve and elaborate for the admiration of the world their novel and wonderful theories. There let them consider not merely the formal logic of the situation, but also the practical effect which their doctrines are likely to have upon a practical and many-sided world. Just as nothing is more ennobling or more serviceable for the world than a religion that is at once both pure and reasonable, so nothing is more degrading and more dangerous to intellect and morals than a feeble fanaticism and a religion run mad. This one proposition all men, but especially religious teachers, should remember, that the eternal existence, the eternal wisdom, and the Creator of all things must always, and in every place, be entirely consistent with Himself. The "Father of Lights" must not be charged with either "variableness" or the "shadow of turning."

